# Supplementary material for: Performance analysis of dual-hop mixed RF-FSO systems combined with NOMA
Source: PLoS One. 2024 Dec 20;19(12):e0315123. doi: 10.1371/journal.pone.0315123 (PMC11661647; doi:10.1371/journal.pone.0315123)
Supplement: S1 Appendix — (PDF) [file pone.0315123.s001.pdf]

**Appendix A: Proof of Proposition 1** The outage probability of $D_1$ may be stated using (18); we therefore have:

$$\begin{aligned}\mathcal{P}_{out}^{1} =& 1 - \underbrace{\Pr\left(\gamma_{R,x_2}^{SIC} > \gamma_{th}^{2}, \gamma_{R,x_1}^{ipSIC} > \gamma_{th}^{1}\right)}_{\mathcal{I}_1} \\ & \times \underbrace{\Pr\left(\gamma_{1,x_2}^{SIC} > \gamma_{th}^{2}, \gamma_{1,x_1}^{ipSIC} > \gamma_{th}^{1}\right)}_{\mathcal{I}_2},\end{aligned} \tag{29}$$

Substituting (2), (3), (5) and (6) to (18), $\mathcal{I}_1$ and $\mathcal{I}_2$ can be calculated from the Eq:

$$\begin{aligned}\mathcal{I}_1 =& \Pr\left(\frac{a_2\rho|h_0|^2}{a_1\rho|h_0|^2+1} > \gamma_{th}^{2}, \frac{a_1\rho|h_0|^2}{\chi a_2\rho|h_0|^2+1} > \gamma_{th}^{1}\right) \\ =& \Pr\left(|h_0|^2 > \Theta_2, |h_0|^2 > \Theta_1\right) \\ =& \Pr\left(|h_0|^2 > \Theta_{\max}\right) \\ =& 1 - F_{|h_0|^2}\left(\Theta_{\max}\right),\end{aligned} \tag{30}$$

where $\Theta_1 = \frac{\gamma_{th}^{1}}{\rho\left(a_1 - \chi a_2\gamma_{th}^{1}\right)}$, $\Theta_2 = \frac{\gamma_{th}^{2}}{\rho\left(a_2 - a_1\gamma_{th}^{2}\right)}$ and $\Theta_{\max} = \max\left(\Theta_1, \Theta_2\right)$.

$\mathcal{I}_1$ may be computed using (14) as

$$\mathcal{I}_1 = 1 - \frac{1}{\Gamma\left(\alpha\right)\Gamma\left(\beta\right)} G_{1,3}^{2,1}\left(\xi\Theta_{\max} \left| \begin{matrix} 1 \\ \alpha, \beta, 0 \end{matrix}\right.\right). \tag{31}$$

Substituting (15b) and (16) into (29), $\mathcal{I}_2$ is written as:

$$\begin{aligned}\mathcal{I}_2 =& \Pr\left(\frac{a_2\rho|h_1|^2}{a_1\rho|h_1|^2+d_1^{\delta}} > \gamma_{th}^{2}, \frac{a_1\rho|h_1|^2}{\chi a_2\rho|h_1|^2+d_1^{\delta}} > \gamma_{th}^{1}\right) \\ =& \Pr\left(|h_1|^2 > \Theta_{\max} d_1^{\delta}\right) \\ =& \int_0^{R_1} f_{d_1}\left(x\right)\left[1 - F_{|h_1|^2}\left(\Theta_{\max} x^{\delta}\right)\right] dx \\ =& 2\sum_{s=0}^{m_1-1} \frac{\mu_1^{s}\Theta_{\max}^{s}}{s! R_1^2} \int_0^{R_1} e^{-\mu_1\Theta_{\max} x^{\delta}} x^{\delta s+1} dx.\end{aligned} \tag{32}$$

Using [59, Eq. (3.352.4)] and applying some algebraic manipulation, $\mathcal{I}_2$ is obtained from the Eq:

$$\mathcal{I}_2 = 2\sum_{s=0}^{m_1-1} \frac{\mu_1^{s}\Theta_{\max}^{s}\gamma\left(\vartheta, \mu_1\Theta_{\max}R_1^{\delta}\right)}{s! R_1^2 \delta \mu_1^{\vartheta}\Theta_{\max}^{\vartheta}}, \tag{33}$$

where $\vartheta = \frac{\delta s+2}{\delta}$.

Combining (33) and (31), we obtain (19).
This completes the proof.

**Appendix B: Proof of Proposition 2** Substituting (30) and (7) into (20), we calculate the outage probability of $D_2$ from the Eq:

$$\mathcal{P}_{out}^{2} = 1 - \mathcal{I}_1 + \mathcal{I}_1 \times \left[ 1 - \underbrace{\Pr\left( \frac{a_2 \rho |h_2|^2}{a_1 \rho |h_2|^2 + d_2^{\delta}} < \gamma_{th}^{2} \right)}_{\mathcal{I}_3} \right], \tag{34}$$

where $\mathcal{I}_1$ is given below (30).

Applying some polynomial expansion manipulation, we obtain $\mathcal{I}_3$ as follows:

$$\begin{aligned}
\mathcal{I}_3 =& \Pr\left(\gamma_{2,x_2} > \gamma_{th}^{2}\right) \\
=& \Pr\left(|h_2|^2 > \Theta_2 d_2^{\delta}\right) \\
=& \int_{R_1}^{R_2} f_{d_2}(x) \left[1 - F_{|h_2|^2}\left(\Theta_2 x^{\delta}\right)\right] dx \\
=& \frac{2}{(R_2^2 - R_1^2)} \sum_{s=0}^{m_2-1} \frac{\mu_2^s \Theta_2^s}{s!} \int_{R_1}^{R_2} e^{-\mu_1 \Theta_{\max} x^{\delta}} x^{\delta s + 1} dx.
\end{aligned} \tag{35}$$

Using [59, Eq. (3.381.8)], [**?**, **?**, 59, Eq. (3.381.9)] [59, Eq. (3.381.10)] and applying some algebraic manipulation, we obtain $\mathcal{I}_4$ as follows:

$$\begin{aligned}
\mathcal{I}_3 =& \frac{2}{(R_2^2 - R_1^2)} \sum_{s=0}^{m_2-1} \frac{\mu_2^s \Theta_2^s}{s!} \left[ \int_{0}^{\infty} e^{-\mu_1 \Theta_{\max} x^{\delta}} x^{\delta s + 1} dx \right. \\
=& \left. - \int_{0}^{R_1} e^{-\mu_1 \Theta_{\max} x^{\delta}} x^{\delta s + 1} dx - \int_{R_2}^{\infty} e^{-\mu_1 \Theta_{\max} x^{\delta}} x^{\delta s + 1} dx \right] \\
=& \frac{2}{(R_2^2 - R_1^2)} \sum_{s=0}^{m_2-1} \frac{\mu_2^s \Theta_2^s}{s! \delta \mu_2^{\vartheta} \Theta_2^{\vartheta}} \left[ \gamma\left(\vartheta, \mu_2 \Theta_2\right) + \Gamma\left(\vartheta, \mu_2 \Theta_2\right) \right. \\
& \left. - \gamma\left(\vartheta, \mu_2 \Theta_2 R_1^{\delta}\right) - \Gamma\left(\vartheta, \mu_2 \Theta_2 R_2^{\delta}\right) \right].
\end{aligned} \tag{36}$$

Substituting (36) and (30) into (34), we obtain (21).
This completes the proof.
