# Supplementary material for: Performance analysis of dual-hop mixed RF-FSO systems combined with NOMA
Source: PLoS One. 2024 Dec 20;19(12):e0315123. doi: 10.1371/journal.pone.0315123 (PMC11661647; doi:10.1371/journal.pone.0315123)
Supplement: S2 Appendix — (PDF) [file pone.0315123.s002.pdf]

Appendix B: Proof of Proposition 2 Substituting (30) and (7) into (20), we calculate the outage probability of D_2 from the Eq:

$$\mathcal{P}_{out}^2 = 1 - \mathcal{I}_1 + \mathcal{I}_1 \times \left[1 - \underbrace{\Pr\left(\frac{a_2 \rho |h_2|^2}{a_1 \rho |h_2|^2 + d_2^\delta} < \gamma_{th}^2 \right)}_{\mathcal{I}_3} \right], \tag{34}$$

where $\mathcal{I}_1$ is given below (30).

Applying some polynomial expansion manipulation, we obtain $\mathcal{I}_3$ as follows:

$$\begin{aligned} \mathcal{I}_3 =& \Pr\left(\gamma_{2,x_2} > \gamma_{th}^2\right) \\ =& \Pr\left(|h_2|^2 > \Theta_2 d_2^\delta\right) \\ =& \int_{R_1}^{R_2} f_{d_2}(x) \left[1 - F_{|h_2|^2}\left(\Theta_2 x^\delta\right) \right] dx \\ =& \frac{2}{(R_2^2 - R_1^2)} \sum_{s=0}^{m_2-1} \frac{\mu_2^s \Theta_2^s}{s!} \int_{R_1}^{R_2} e^{-\mu_1 \Theta_{\max} x^\delta} x^{\delta s + 1} dx. \end{aligned} \tag{35}$$

Using [59, Eq. (3.381.8)], [59, Eq. (3.381.9)], [59, Eq. (3.381.10)] and applying some algebraic manipulation, we obtain $\mathcal{I}_4$ as follows:

$$\begin{aligned} \mathcal{I}_3 =& \frac{2}{(R_2^2 - R_1^2)} \sum_{s=0}^{m_2-1} \frac{\mu_2^s \Theta_2^s}{s!} \left[\int_0^\infty e^{-\mu_1 \Theta_{\max} x^\delta} x^{\delta s + 1} dx \right. \\ =& \left. - \int_0^{R_1} e^{-\mu_1 \Theta_{\max} x^\delta} x^{\delta s + 1} dx - \int_{R_2}^\infty e^{-\mu_1 \Theta_{\max} x^\delta} x^{\delta s + 1} dx \right] \\ =& \frac{2}{(R_2^2 - R_1^2)} \sum_{s=0}^{m_2-1} \frac{\mu_2^s \Theta_2^s}{s! \delta \mu_2^\vartheta \Theta_2^\vartheta} \left[\gamma\left(\vartheta, \mu_2 \Theta_2\right) + \Gamma\left(\vartheta, \mu_2 \Theta_2\right) \right. \\ & \left. - \gamma\left(\vartheta, \mu_2 \Theta_2 R_1^\delta\right) - \Gamma\left(\vartheta, \mu_2 \Theta_2 R_2^\delta\right) \right]. \end{aligned} \tag{36}$$

Substituting (36) and (30) into (34), we obtain (21).
This completes the proof.
