# Supplementary material for: Performance analysis of dual-hop mixed RF-FSO systems combined with NOMA
Source: PLoS One. 2024 Dec 20;19(12):e0315123. doi: 10.1371/journal.pone.0315123 (PMC11661647; doi:10.1371/journal.pone.0315123)
Supplement: Attachment — Submitted filename: Response_letter.pdf [file pone.0315123.s004.pdf]

Response to Editor's and Reviewers' Comments of Paper PONE-D-24-24546:

Performance analysis of dual-hop mixed RF-FSO systems combined with NOMA

Addressed Comments for possible publication in
PLOS ONE

Tran Cong Hung, et.al

November 3, 2024

November 3, 2024

Dear Editor-in-Chief,

This is the revised version of the previous submission with paper ID PONE-D-24-24546. Thank you very much for handling the review process of our paper. We appreciate your time and effort invested in reviewing the manuscript.

In this revision, all comments and suggestions of reviewers have been carefully considered and are incorporated where appropriate. In addition, we have provided detailed answers to the comments and questions raised by the reviewers. Unless otherwise stated, all the numbered items (figures, equations, references, etc.) in this response letter refer to those in the revised manuscript.

Yours sincerely,
Tran Cong Hung, et.al

Note: To help the legibility of the remainder of this response letter, all the reviewers' comments and questions are typeset in italic font. Our responses and remarks are written in plain font. Rephrase sentences are typeset in blue color.

PLOS ONE
Paper ID PONE-D-24-24546
Authors' Response to Reviewer 1's Comments

First of all, we sincerely thank you for reading our manuscript and providing us with constructive comments. Based on your comments, we have revised our manuscript accordingly and provided our detailed responses to your comments as follows:

This study considered the Performance Analysis of Dual-Hop Mixed RF-FSO Systems Combined with NOMA. In general, this paper is well-written, and I thoroughly enjoyed reading it. It has a limitation of contribution compared with previous papers. Some weak points need to be addressed:

Comment 1:

In this paper, the author considers two users. How about multiple users?

Author's response

We would like to thank you for your careful reviewing and constructive suggestion. In this manuscript, we focus on the homogeneous PPPs for user to evaluate the performance. Thus, we consider two basic users in NOMA as [R1,R2] to analyze the performance in the proposed model. For the multi-user case, it is very complicated to apply in the propsed model so we will consider them in future manuscript.
[R1]: Y. Ye, Y. Li, D. Wang and G. Lu, "Power splitting protocol design for the cooperative NOMA with SWIPT," 2017 IEEE International Conference on Communications (ICC), Paris, France, 2017, pp. 1-5, doi: 10.1109/ICC.2017.7996751
[R2]: D. -T. Do, C. -B. Le, A. Vahid and S. Mumtaz, "Antenna Selection and Device Grouping for Spectrum-Efficient UAV-Assisted IoT Systems," in IEEE Internet of Things Journal, vol. 10, no. 9, pp. 8014-8030, 1 May1, 2023, doi: 10.1109/JIOT.2022.3229592.

Comment 2:

The authors should compare your work with the case of OMA.

Author's response

Thank you for your careful reviewing and constructive comments. To address this concern, we have updated and highlighted Figure 1 (Figure 6 in the revised manuscript) to compare our work with OMA. In the revised manuscript, we have added the sentence such as:
In Fig. 6 presents the outage probability versus PS (in dBm) for different scenarios in a wireless communication system, showing that the NOMA scheme achieves better performance for both near and far users compared to the conventional OMA, with asymptotic and simulated results depicted by dashed and solid lines, respectively, demonstrating the superior robustness of NOMA, especially at higher transmit power levels.

Comment 3:

How do the power allocation factors influence the system performance of the considered system

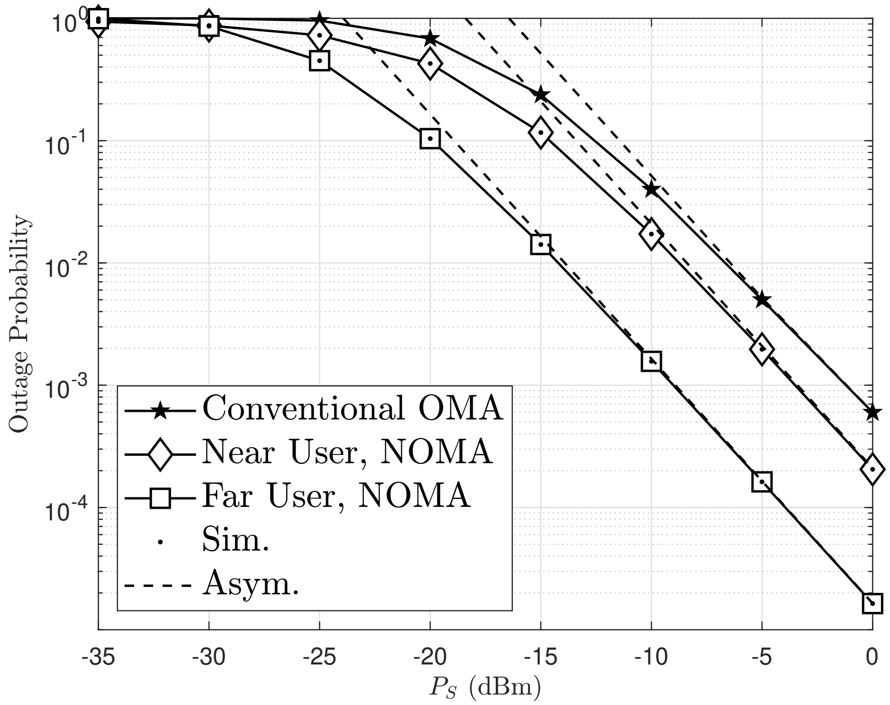

Figure 1: Outage probability versus P_S in NOMA and OMA, with $m = 2$, $a_1 = 0.1$ and $a_2 = 0.9$.

Author's response

We would like to thank you for your comment. In the revised manuscript, we have updated Figure 2 (Figure 4 in the revised manuscript) to show the impact of the power allocation factors. In particular, we have updated and highlighted the sentence as:
Fig. 4 indicates that two users experience different outage performance as the power allocation factor varies. Specifically in this case, the lowest OP for user D_1 occurs when $a_2 = 0.6$, while for user D_2, it occurs when $a_2 = 0.95$. This behavior can be explained by considering that as a_2 is increases, a_1 decreases, leading to an increase in the received SINR at D_2, as indicated by Equation (7). Hence, the OP at the D_2 source always decreases. However, the received SINR at D_1 may increase (based on Equation (5)) or decrease (based on Equation (6)). Therefore, the OP expression in Equation (18) does not show a clear trend of increasing or decreasing, but through simulation and under the condition $a_2 > a_1$, when a_2 increases from 0.6 to 0.95, the OP of near users also increases and converges to an asymptotic value when $a_2 = 0.95$. As a result, as a_2 increases, the far user's OP performance improves while the near user's OP performance deteriorates, and vice versa. To ensure fairness between users, an optimal level of the power allocation factor exists; this is achieved when $a_2 \approx 0.7$ (corresponding to $a_1 = 0.3$), resulting in nearly equal OP performance for both near and far users.

Comment 4:

What is the CSI assumption of the article, please describe it in a real scenario.

Author's response

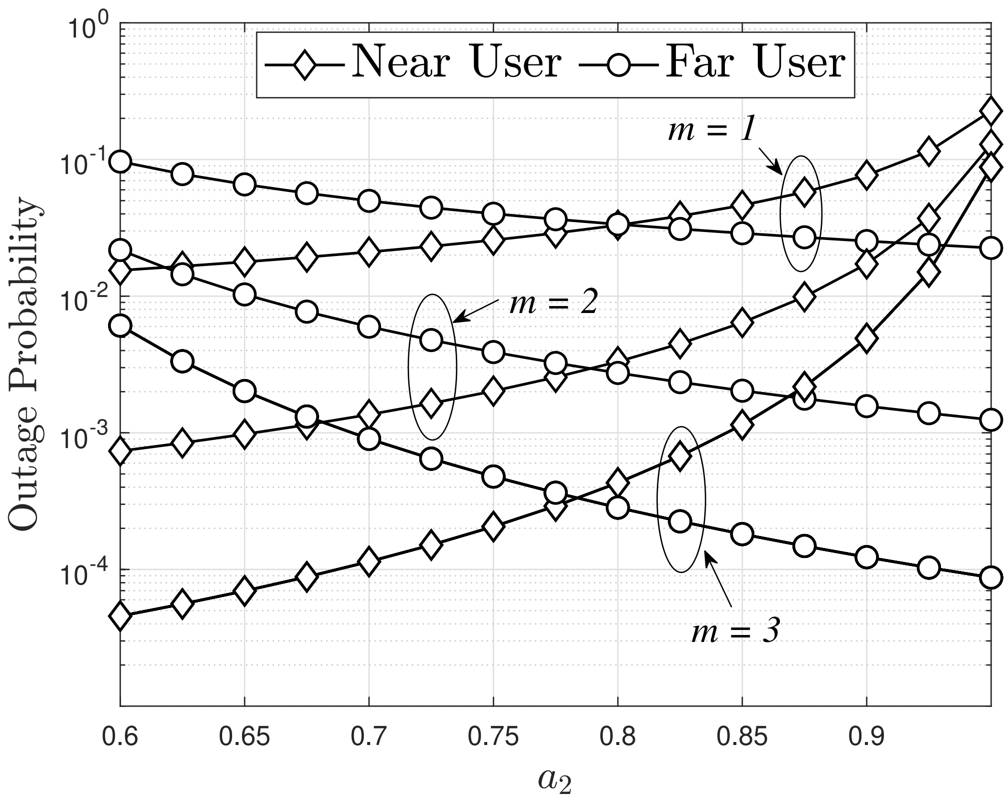

Figure 2: Outage probability versus power allocation factors, with $\rho = 30$ [dB], $R_{th}^1 = 1$ and $R_{th}^2 = 0.5$.

We would like to thank you for your constructive suggestion. In this manuscript, we consider the S and R has perfect knowledge about the channel state information (CSI) to detect the signal. In the revised manuscript, we have added the sentence to improve the presentation as:
It is assumed that R operates in half-duplex mode and that the channel state information (CSI) is perfect at each terminal to detect the signal

Comment 5:

The presentation of the whole paper is poor. We need more improvements.

Author's response

Thank you for your constructive comments. We have used professional proofreading to revise and improve the presentation for the manuscript.

Finally, special thanks to you for your constructive comments. We tried our best to improve the manuscript and appreciate the Editor's warm work earnestly, and hope that the correction will meet with approval. Once again, thank you very much for your comments and suggestions.

PLOS ONE
Paper ID PONE-D-24-24546
Authors' Response to Reviewer 2's Comments

First of all, we sincerely thank you for reading our manuscript and providing us with constructive comments. Based on your comments, we have revised our manuscript accordingly and provided our detailed responses to your comments as follows:

Comment 1:

The introduction is so general. The topic of the paper is about FSO and NOMA. The general discussion should be removed, and the related literature should be included. The current challenges should be cited from the papers, which the authors would address in the rest of the paper. The given references could also be helpful:
-https://opg.optica.org/ol/fulltext.cfm?uri=ol-48-17-4548&id=536662
- https://www.mdpi.com/2304-6732/10/10/1073
- https://opg.optica.org/ol/abstract.cfm?uri=ol-48-15-4101
- https://www.sciencedirect.com/science/article/abs/pii/S1434841123005587?via%3Dihub

Author's response

We would like to thank you for your careful reviewing and constructive suggestion. To address this concern of Reviewer, we firstly remove some general discussion. Secondly, we have added the paragraph to decrible the FSO-RF NOMA system. Finally, we have updated Table 1 to show the comparation and contribution of our work with related work. In particular, we have updated in the revised manuscript as:
- In page 3
In addition, the incorporation of NOMA with mixed FSO-RF networks to achieve better communication performance [43-48]. A two-hop uplink NOMA RF-FSO network was investigated in [43] in order to meet the high throughput requirements for the backhaul connection. The closed-form formulation of OP and ergodic channel capacity under various interference situations was also provided. The subject of the combined optimization of power allocation and harvesting time was further investigated in [44] when the analytical expressions of OP and throughput were taken into consideration in the context of an energy harvesting enabled mixed FSO-RF network. By including NOMA into both the FSO and RF connections, the authors of [45] suggested a down-link FSO-RF network and determined the closed-form expression of OP for every user. The analytical formulation of OP was introduced in [46], taking into account that the RF channel has a Rayleigh distribution and the FSO channel has an M-distribution. [47] provided the analytical and asymptotic formulas of secrecy OP (SOP) for an FSORF system with NOMA while accounting for the imperfect channel state information. For a NOMA-based hybrid RF-UWOC system, the asymptotic formulations of ergodic capacity and the closed-form formulas of OP were provided in [48].
- In page 3 and 4
Table 1 shows the comparison between our work and related papers and the key notations are used in this paper, as shown in Table 2

Comment 2:

Table I: The comparison of our work and the previous works.

Ref./Prop.	Dual-hop FSO-RF	Nakagami-m fading	NOMA	OP	Asymptotic	Stochastic Geometry
[49]	✓	✓	✓	✓	X	X
[50]	✓	✓	✓	✓	✓	X
[51]	✓	✓	X	✓	X	X
[52]	X	X	X	✓	X	✓
[53]	✓	X	X	✓	✓	X
Our study	✓	✓	✓	✓	✓	✓

Many of the acronyms are not repeated in the abstract, so it is better to remove them.

Author's response

Thank you for your careful reviewing and constructive comments. In the revised manuscript, we have removed the non-repeated acronyms to improve the presentation of the manuscript. In particular, we have updated the abtract as:
This paper investigates the performance of hybrid radio frequency/free space optical (RF/FSO) systems combined with non-orthogonal multiple access communications technology. We examine a scenario where the source and destination are separated by a large distance, with no direct link between them. The relay, denoted R, operates using the decode-and-forward (DF) protocol. Under the DF relaying scheme, the relay employs successive interference cancellation (SIC). In this setup, the FSO link from the source to the relay follows a Gamma-Gamma distribution, while the RF links from the relay to multiple users follow a Nakagami-m distribution. Based on this system model, we analyze the outage probability (OP). Our findings indicate a direct relationship between SIC and OP performance: the higher the SIC capability, the more effective the system. In addition, the system's performance is dependent on the parameters of the FSO channel. Finally, Monte Carlo simulations are presented to further validate our framework and findings.

Comment 3:

Details about NOMA and its working principal is missing. I) how will it work in FSO system, ii) what will be/could be the transmission range.

Author's response

We would like to thank you for your careful reviewing and constructive suggestion. In this manuscript, we have updated and highlighted the sentences to describe the NOMA principle for FSO link, and RF link to clarify the application of NOMA principle for the manuscript. Furthermore, we consider the distance from S to R is 1000 m and the distance of D_i based on homogeneous poisson point process with circle radius R_i [R3]. In the revised manuscript, we have revised and highlighted the sentences as:
- In page 4:
"In the first time slot, by applying NOMA principle, S using NOMA signaling $\sqrt{a_1 P_S} x_1 + \sqrt{a_2 P_S} x_2$ to transmits to R through the FSO link, where P_S is the transmission power at S, x_1 and x_2 are the unit-power data symbols, a_1 and a_2, $a_2 > a_1$ are the corresponding power allocation coefficients. The signal received at the R is therefore expressed as"

- In page 5:
"By similarly applying NOMA principle in FSO link, R transmit the NOMA signaling $\sqrt{a_1 P_R} x_1 + \sqrt{a_2 P_R} x_2$ to user D_i"
- In page 6:
"Using the NOMA protocol, since the user with the highest received signal strength, D_1, applies SIC, the SINRs required to perform SIC for x_1 and x_2 at D_1 are given by"

Comment 4:

It is strongly claimed by the authors: This paper provides a performance analysis of a dual-hop mixed RF-FSO system integrated with NOMA communications technology. While in the paper after section 2, I don't see any evidence/contribution of the authors about NOMA, they have discussed numerically FSO and RF in details, but nothing is shown/presented about NOMA.

Author's response

We would like to thank you for your careful reviewing and constructive suggestion. In this manuscript, we have applied NOMA to two links FSO and RF. Specifically, NOMA principle focuses on power allocation to users to decode the user's signal. Furthermore, the power allocation will be based on the distance or channel quality to meet the QoS. In the numerical results section, we present Figure 4 to show the impact of the power allocation factor on the user's OP. We can easily see the impact of the power allocation factor (NOMA) on the user's performance. We further updated Figure 6 to compare NOMA and OMA. It can be observed as the performance of NOMA is better than OMA. For the NOMA principle, we have added the sentence as Comment 3, and Figure 6 have updated as in Comment 3 of Reviewer 1.

Comment 5:

How does the Hybrid FSO+NOMA work? i) this point is not discussed, ii) similarly, the numerical+theoretical details are missing, and iii) results related to this claim are not presented clearly. I would suggest the authors to revise the paper in detail.

Author's response

Thank you for your careful reviewing and constructive comments. Firstly, for the Hybrid FSO-NOMA, we have applied the NOMA principle to FSO network to serve multiple users in the same spectrum. Secondly, we have updated Figure 3 (Figure ... in the revised manuscript) to compare the performance of mixed FSO-RF NOMA network and FSO NOMA network. Thirdly, we have added the sentence in the Nummerical Result section to present the result of the performance of FSO NOMA and mixed FSO-RF NOMA.
- In page 3
Exploiting the advantages of both the high data rates facilitated by FSO systems and the high spectral efficiency introduced by NOMA serves as the primary motivation for combining FSO and NOMA. Another reason is that NOMA typically operates well in situations where the power received from various transmitters varies significantly [28].
- In page 13
Fig. 3 shows the outage probability of FSO and FSO-RF links at both near and far users as a function of P_S(dBm), with simulated (solid lines) and asymptotic (dashed lines) results indicating better performance at higher power levels. the figure highlights that both user distance and link

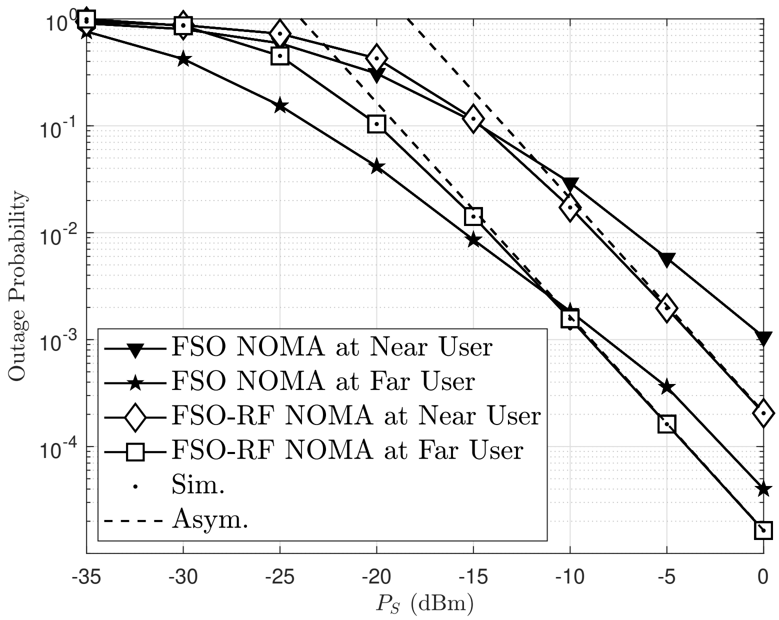

Figure 3: Outage probability versus P_S in FSO link and FSO-RF link, with $m = 2$ and $\chi = 0.01$.

type (FSO or FSO-RF) significantly impact outage probability, with the near-user FSO link providing the best reliability. Increasing transmit power P_S reduces the outage probability across all configurations, the performance of users for FSO-RF NOMA network is better than FSO NOMA network.

Finally, special thanks to you for your constructive comments. We tried our best to improve the manuscript and appreciate the Editor's warm work earnestly, and hope that the correction will meet with approval. Once again, thank you very much for your comments and suggestions.
